# Increasing conversion efficiency of two-step photon up-conversion solar cell with a voltage booster hetero-interface

**DOI:** 10.1038/s41598-018-19155-x

**Published:** 2018-01-17

**Authors:** Shigeo Asahi, Kazuki Kusaki, Yukihiro Harada, Takashi Kita

**Affiliations:** Department of Electrical and Electronic Engineering, Graduate School of Engineering, Kobe University, 1-1 Rokkodai, Nada, Kobe 657-8501 Japan

## Abstract

Development of high-efficiency solar cells is one of the attractive challenges in renewable energy technologies. Photon up-conversion can reduce the transmission loss and is one of the promising concepts which improve conversion efficiency. Here we present an analysis of the conversion efficiency, which can be increased by up-conversion in a single-junction solar cell with a hetero-interface that boosts the output voltage. We confirm that an increase in the quasi-Fermi gap and substantial photocurrent generation result in a high conversion efficiency.

## Introduction

In the face of global environmental issues, solar energy is attracting considerable interest as a renewable energy source. The energy conversion efficiency of a single-junction solar cell (SJSC) obeys the so-called Shockley–Queisser limit, taking into consideration various losses caused by light transmission and absorbed excess energy resulting in the upper limit of approximately 31% under one-sun illumination^1^. A SJSC made of a wide-gap material suffers from a large transmission loss and exhibits a small photocurrent, despite having a high output voltage. Conversely, an SC with a small bandgap semiconductor exhibits a large photocurrent; however, the output voltage is small. This trade-off relation against the bandgap gives rise to the conversion limit^2^. To surpass this limit, a multi-bandgap system comprising different bandgap semiconductors is a promising method to reduce the loss^3,4^. Multi-junction SCs, which are currently the best SC structure attaining the world record of conversion efficiency^5^, possesses a stacked structure of SJSCs with different bandgaps covering a wide solar spectrum, in which excited carriers can transport through the tunnel junctions formed between the SC diodes. The output voltage of the series junctions becomes high and the photocurrent increases. Another method of utilizing the multi-bandgap system is through an intermediate band (IB) SC^6–9^, in which additional bands inserted in the bandgap can absorb below-gap photons corresponding to transitions from the valence band (VB) to the IB and from the IB to the conduction band (CB), as well as the ordinal interband absorption from the VB to the CB. IBSCs are a promising SC device for realising ultrahigh conversion efficiencies greater than 63% under maximum concentration and 47% even under one-sun illumination^6^. In IBSCs, an electron excited in the IB pumped out to the CB upon absorbing a below-gap photon. Thus, the potential energy of the excited electron is up-converted by this two-step process, resulting in an increase in photocurrent without reducing the output voltage. However, in general, the transition strength between the IB and CB is very weak^10^ because of not only the optical selection rule but also a relatively short electron lifetime at the IB. The intraband absorption strength is proportional to the oscillator strength and the carrier density in the IB. According to the optical selection rule for the intraband transition, three dimensional systems such as bulk and quantum dots are promising. In the case of quantum dots, the oscillator strength of the intraband transition is proportional to the quantum-dot density. Generally, the density is not so high^11^. Besides, the shape of self-assembled quantum dots is generally disc like^11^ which reduces the oscillator strength for excitation with an in-plane polarization. The carriers confined in the quantized state (the IB) quickly recombine, and, thereby, the electron lifetime becomes short. In particular, that is significant in the type I quantized state of InAs/GaAs quantum dots^12,13^. On top of that, thermal carrier excitation reduces the electron density in the IB. Thus, in general, the transition strength between the IB and CB is weaken by both the oscillator strength and the electron density in the IB. The type II quantum confined system is one of promising candidates to extend the electron lifetime in the IB. Some materials have been proposed and investigated^14–17^. However, the interband transition in the type II quantum confined system, is spatially indirect, and, thereby, the interband-oscillator strength is lower than that of the type I quantum confined system. In addition, excited electrons in the CB quickly relax to the IB^18^. According to the theoretical simulation performed by Tomić, non-radiative relaxation time of electrons in the CB into the IB is few picoseconds for the quantum-dot IBSCs^19^. This fast process is unavoidable and also gives rise to reduction of the conversion efficiency.

If we can spatially draw the IB out of the bandgap, electrons excited in the CB will be prevented from relaxing to the IB. Recently, we proposed a two-step photon up-conversion (TPU)-SC^20^ realising the ratcheting process^21–23^ using a hetero-interface comprising different semiconductor bandgaps. In the case of a TPU-SC fabricated on a *p*-type substrate, below-gap photons passing through the wide bandgap semiconductor (WGS) layer excite the narrow bandgap semiconductor (NGS), in which excited electrons drift towards the WGS/NGS hetero-interface and accumulate there, while holes reach the *p*-layer. The long-lived electron, which was prevented from recombining at the hetero-interface is then efficiently excited by another below-gap photon in the NGS and lifted to the CB of the WGS. This TPU boosts the output voltage at the hetero-interface. In the TPU-SC, the two-step excitation in our device does not produce a high-energy photon but creates a free carrier in the CB. The potential energy of the CB is far from each excitation photon energy. Using two photons, an electron is finally pumped from the VB to the CB by way of the CB of the NGS. Here, the carrier energy is up-converted by two photons, and the excitation from the CB to the VB is equivalent to an event caused by a high-energy photon.

In this work, we study the increase in conversion efficiency of a TPU-SC with a voltage booster hetero-interface in the detailed-balance framework, considering a steady state between the carrier generation and recombination at the optimum operation point of an SC. We clarify the band discontinuity effects on the conversion efficiency between the NGS and WGS and offer a route to high-conversion-efficiency solar cells exceeding 50%.

## Results

### Concept of TPU-SC and model used in calculation

**Figure 1:**
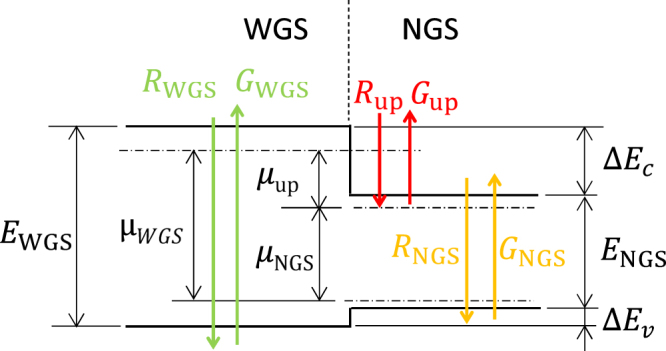
Schematic calculation model of TPU-SC. *E*_WGS_ and *E*_NGS_ are the bandgaps of the WGS and NGS, respectively. *μ*_WGS_ and *μ*_NGS_ are the quasi-Fermi level separations in the WGS and NGS, respectively, and *μ*_up_ is the quasi-Fermi level separation due to TPU. Δ*E*_c_ and Δ*E*_v_ are the CB and VB discontinuity, respectively. *G*_WGS_ and *G*_NGS_ are the carrier-generation rates in the WGS and NGS, respectively, and *G*_up_ is the carrier-generation rate due to up-conversion. *R*_WGS_, *R*_NGS_, and *R*_up_ are the carrier-recombination rates in the WGS, NGS, and at the hetero-interface, respectively.

Figure 1 shows a schematic band diagram of a typical TPU-SC. The TPU-SC is composed of a single-diode structure containing a hetero-interface which consists of a WGS and NGS. It is noted that the IB in this TPU-SC is the CB of the NGS. Sunlight irradiates the WGS side (left-hand side of Fig. 1), high-energy photons are absorbed in the WGS layer, and excited electrons and holes drift in opposite directions towards the *n*- and *p*-type electrodes, respectively. In the case of a TPU-SC fabricated on a *p*-type substrate, below-gap photons passing through the WGS layer excite the NGS, in which excited electrons drift towards the WGS/NGS hetero-interface and accumulate there, while holes reach the *p*-layer. The spatial carrier separation prevents the recombination of electrons with holes and extends the electron lifetime. Electrons with longer lifetimes have a greater potential for intraband absorption in the TPU because the oscillator strength of the second photon absorption is proportional to the electron density in the initial state of the second transition. One appealing advantage for TPU-SCs compared with conventional IBSCs is the small spatial overlap of the quasi-Fermi level of electrons in the NGS and WGS. Generally, the quasi-Fermi level of the IB spatially overlaps with the quasi-Fermi level of the CB, causing the IB to thermally couple with the CB, resulting in a reduction in the output voltage. In contrast to such conventional IBCSs, the hetero-interface which boosts the voltage in a TPU-SC is separated from the portion generating additional current by absorbing below-gap photons in the WGS. This structure prevents energy relaxation from the CB of the WGS to that of the NGS.

Our calculation is based on the detailed-balance framework which was originally proposed by Shockley and Queisser in ref.^1^. As described in ref.^1^, the detailed-balance framework considers a steady state between the carrier generation and recombination at the optimum operation point of a SC. This model has been widely used to calculate the ideal conversion efficiency of a SC. Here, we ignore nonradiative processes in the SC for predicting the ideal conversion efficiency limit. The total photon emission flux, *N*, with the energy range between *E*_min_ and *E*_max_ can be calculated using the generalised Planck equation incorporating the effect of the chemical potential, *μ*^4,24^:

1 $$N({E}_{{\rm{\min }}},\,{E}_{{\rm{\max }}},\,T,\,\mu )=\frac{2\pi }{{h}^{3}{c}^{2}}{\int }_{{E}_{{\rm{\min }}}}^{{E}_{{\rm{\max }}}}\frac{{E}^{2}}{\exp \{(E-\mu )/{k}_{b}T\}-1}{\rm{d}}E,$$

where *T* is the temperature, *h* is Planck’s constant, *c* is the speed of light, and *k*_*b*_ is Boltzmann’s constant. By using equation (1), the generation rates of *G*_WGS_ in the WGS, *G*_NGS_ in the NGS, and *G*_up_ for TPU can be expressed by:

2 $${G}_{{\rm{WGS}}}=X{f}_{{\rm{sun}}}N({E}_{{\rm{WGS}}},\,\infty ,\,{T}_{{\rm{sun}}},\,0)+(1-X{f}_{{\rm{sun}}})N({E}_{{\rm{WGS}}},\,\infty ,\,{T}_{{\rm{cell}}},\,0),$$

3 $${G}_{{\rm{NGS}}}=X{f}_{{\rm{sun}}}N({E}_{{\rm{NGS}}},\,{E}_{{\rm{WGS}}},\,{T}_{{\rm{sun}}},\,0)+(1-X{f}_{{\rm{sun}}})N({E}_{{\rm{NGS}}},\,{E}_{{\rm{WGS}}},\,{T}_{{\rm{cell}}},\,0),$$

and

4 $${G}_{{\rm{up}}}=X{f}_{{\rm{sun}}}N(\Delta {E}_{c},\,{E}_{{\rm{NGS}}},\,{T}_{{\rm{sun}}},\,0)+(1-X{f}_{sun})N(\Delta {E}_{c},\,{E}_{{\rm{NGS}}},\,{T}_{{\rm{cell}}},\,0),$$

where *X* is the solar concentration factor, ${f}_{{\rm{sun}}}=2.16\times {10}^{-5}$ is the solid angle of the sun, ${T}_{{\rm{sun}}}=\mathrm{6,000}\,{\rm{K}}$ is the temperature of sun, *E*_WGS_ and *E*_NGS_ are the bandgap energies of the WGS and NGS, respectively, *ΔE*_c_ is the CB discontinuity between the WGS and NGS, and ${T}_{{\rm{cell}}}=300\,{\rm{K}}$ is the temperature of the SC. Here, we considered that *G*_up_ takes place solely within the NGS and then the carriers are transferred to the WGS. However, in the practical TPU-SC, the intraband excitation of the wavefunction component of accumulated electrons penetrating into the WGS barrier is strong. Conversely, the wavefunction component located in the NGS region should be solely excited within it, and the carriers are transferred to the WGS in the internal electric field. The relation between *E*_WGS_, *E*_NGS_, Δ*E*_c_, and VB discontinuity, Δ*E*_v_, can be given by:

5 $${E}_{{\rm{WGS}}}={E}_{{\rm{NGS}}}+{\rm{\Delta }}{E}_{{\rm{c}}}+\Delta {E}_{{\rm{v}}},$$

Each recombination rate can be given by:

6 $${R}_{{\rm{WGS}}}=N({E}_{{\rm{WGS}}},\,\infty ,\,{T}_{{\rm{cell}}},\,{\mu }_{{\rm{WGS}}}),$$

7 $${R}_{{\rm{NGS}}}=N({E}_{{\rm{NGS}}},\,{E}_{{\rm{WGS}}},\,{T}_{{\rm{cell}}},\,{\mu }_{{\rm{NGS}}}),$$

and:

8 $${R}_{{\rm{c}}}=N({\rm{\Delta }}{E}_{{\rm{c}}},\,{E}_{{\rm{NGS}}},\,{T}_{{\rm{cell}}},\,{\mu }_{{\rm{up}}}),$$

where *μ*_WGS_ and *μ*_NGS_ are the quasi-Fermi level separations in the WGS and NGS, respectively, and *μ*_up_ is the quasi-Fermi level separation due to TPU (see Fig. 1). We do not consider the surface recombination at the WGS/NGS hetero-interface in this study. Here, we take into consideration electrons accumulated at the hetero-interface and the TPU occurring in the CB of the NGS. A similar process transpires when holes are accumulated at the hetero-interface and TPU occurs in the VB. In this case, *ΔE*_c_ in equations (7) and (8) is replaced by *ΔE*_v_. According to these relations, the total current, *J*, generated in the TPU-SC can be obtained by:

9 $$\frac{J}{q}={G}_{{\rm{WGS}}}+{G}_{{\rm{NGS}}}-{R}_{{\rm{WGS}}}-{R}_{{\rm{NGS}}},$$

where *q* is the electronic charge. In the TPU-SC, the following current matching condition of TPU must be satisfied:

10 $$0={G}_{{\rm{NGS}}}+{G}_{{\rm{up}}}-{R}_{{\rm{NGS}}}-{R}_{{\rm{up}}}.$$

The output voltage of the TPU-SC is given by

11 $$qV={\mu }_{{\rm{WGS}}}={\mu }_{{\rm{NGS}}}+{\mu }_{{\rm{up}}}.$$

Finally, the total electrical power generated in TPU-SC is calculated as a product of *VJ* and, hence, the expected conversion efficiency can be estimated by the division of *VJ* by the total incident photon energy.

### Conversion efficiency and recombination rate

**Figure 2:**
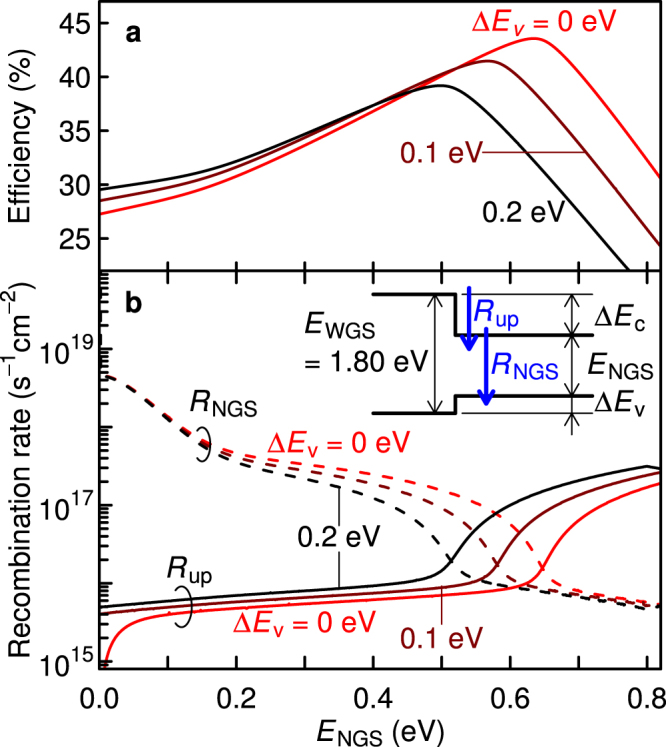
Detailed balance calculation for TPU-SC as a function of NGS bandgap (*E*_NGS_) under one-sun illumination. (**a**) Conversion efficiency and (**b**) recombination rate in NGS (*R*_NGS_, dashed lines) and CB discontinuity (*R*_up_, solid lines). The bandgap of the WGS *E*_WGS_ is fixed at 1.80 eV. The inset of Fig. 2b shows an illustration of the band diagram.

We calculated the conversion efficiency as a function of the NGS bandgap, *E*_NGS_, for different VB discontinuities, Δ*E*_v_. Figure 2a presents the results under one-sun illumination. Here, the bandgap of WGS, *E*_WGS_, is assumed to be 1.8 eV. As Δ*E*_v_ is fixed in this calculation, Δ*E*_c_ decreases with *E*_NGS_. When *E*_NGS_ is 0 eV, the efficiency becomes equivalent to that of the SJSC with the same bandgap as Δ*E*_c_ because Δ*E*_v_ corresponds to a voltage loss. The efficiency increases with *E*_NGS_ because of the voltage boost accomplished by TPU at the hetero-interface. Then, the conversion efficiency reaches a maximum of 43.6% at an *E*_NGS_ of 0.63 eV when Δ*E*_v_ = 0. This value coincides with the efficiency calculated for the well-known ideal IBSC with the same bandgap of 1.8 eV for the host semiconductor. The value of *E*_NGS_, at which the conversion efficiency exhibits the peak, shifts with varying Δ*E*_v_. An increasing Δ*E*_v_ leads to a monotonic decrease in the peak efficiency because Δ*E*_v_ leads to a voltage loss at the hetero-interface. This indicates that a zero-band discontinuity (Δ*E*_v_ = 0) achieves a maximum conversion efficiency. Figure 2b shows detailed recombination rates, *R*_NGS_, for the interband transition in the NGS and energy relaxation rates, *R*_up_, for the intraband transition at the hetero-interface. When *E*_NGS_ is small, *R*_NGS_ is higher than *R*_up_ by several orders of magnitude. As *E*_NGS_ increases, *R*_NGS_ decreases while *R*_up_ increases and, finally, *R*_up_ exceeds *R*_NGS_. When *R*_up_ and *R*_NGS_ coincide, the efficiency exhibits a peak. After reaching the maximum efficiency, *R*_up_ becomes remarkably high, causing the efficiency to rapidly drop.

### Effect of sunlight concentration and valence-band discontinuity on voltage boost

**Figure 3:**
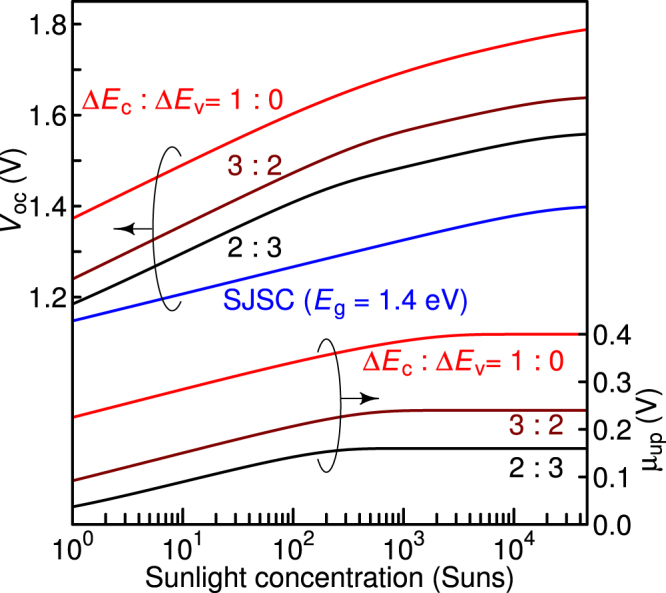
Calculated open-circuit voltage (*V*_oc_) and quasi-Fermi separation at the hetero-interface (*μ*_up_) and the open-circuit conditions as a function of sunlight concentration at different ratios of Δ*E*_c_ to Δ*E*_v_. The blue line indicates the *V*_oc_ of a single-junction solar cell (SJSC) with a bandgap energy (*E*_g_) of 1.4 eV. In this calculation, the bandgap energies of WGS and NGS are fixed at 1.8 and 1.4 eV, respectively.

It is well known that concentrator photovoltaics are one of the promising photovoltaic technologies which improve the conversion efficiency. Figure 3 displays the calculated results of the open-circuit voltage, *V*_oc_, as a function of sunlight concentration at various Δ*E*_c_-to-Δ*E*_v_ ratios. Here, we choose *E*_WGS_ and *E*_NGS_ of 1.8 and 1.4 eV, respectively. In Fig. 3, the slopes of *V*_oc_ for the TPU-SC are slightly steeper than that of the SJSC (blue line) at lower sunlight concentrations, which arises from the voltage-boost effect caused by TPU. At higher concentrations, all slopes coincide with that of the SJSC. The calculated quasi-Fermi splitting, μ_up_, at the hetero-interface at each Δ*E*_c_-to-Δ*E*_v_ ratio is also shown in Fig. 3. With the increase in solar concentration, μ_up_ increases and finally reaches Δ*E*_c_. As shown in Fig. 3, once μ_up_ saturates, the slope of *V*_oc_ becomes small. At lower concentrations, the increases in μ_NGS_ and μ_up_ contribute to the increase in *V*_oc_. Conversely, at higher concentrations, only the increase in μ_NGS_ drives the increase in *V*_oc_, resulting in the shallow slope. The short-circuit current, on the other hand, completely coincides with that of the SJSC with a bandgap of 1.4 eV for any Δ*E*_c_-to-Δ*E*_v_ ratio, as the current in the TPU-SC band lineup is limited by the carrier generation rate in the NGS.

**Figure 4:**
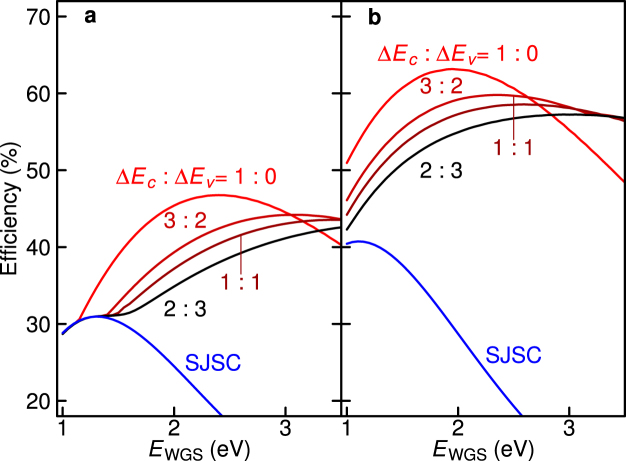
Calculated efficiency as a function of *E*_WGS_ at various Δ*E*_c_-to-Δ*E*_v_ ratios. (**a**) Under one-sun illumination and (**b**) maximum concentration. *E*_NGS_ is optimised to maximise the efficiency under the condition that *E*_NGS_ is always below *E*_WGS_ (*E*_NGS_ < *E*_WGS_). The blue lines indicate the efficiency of SJSC with the bandgap of *E*_WGS_.

Next, we focus on the effect of VB discontinuity on thes conversion efficiency. We systematically calculated the efficiency at different Δ*E*_c_-to-Δ*E*_v_ ratios. Figure 4a,b present the results as a function of *E*_WGS_ under one-sun and maximum concentration, respectively. In this calculation, *E*_NGS_ is optimised to maximise the efficiency when calculating the value at each *E*_WGS_. When *E*_WGS_ is small, Δ*E*_c_ becomes small, and the calculated efficiency coincides with that of the SJSC indicated by the blue line, because the voltage boost effect becomes small in such a small band discontinuity. The efficiency of the TPU-SC increases with *E*_WGS_. Then, the calculated efficiency curve reaches a maximum. With the increase in Δ*E*_v_, the optimum *E*_WGS_ exhibiting the maximum increases and its peak efficiency decreases. Here, it is noted that the efficiency curve given at Δ*E*_v_ = 0 completely coincides with the result for an ideal IBSC proposed in ref.^6^. As the increase in Δ*E*_v_ causes voltage loss at the hetero-interface, the efficiency decreases and, therefore, the optimum *E*_WGS_ tends to become wide, improving the role of the voltage boost.

### Optimum bandgap energies of WGS and NGS for maximising the conversion efficiency

**Figure 5:**
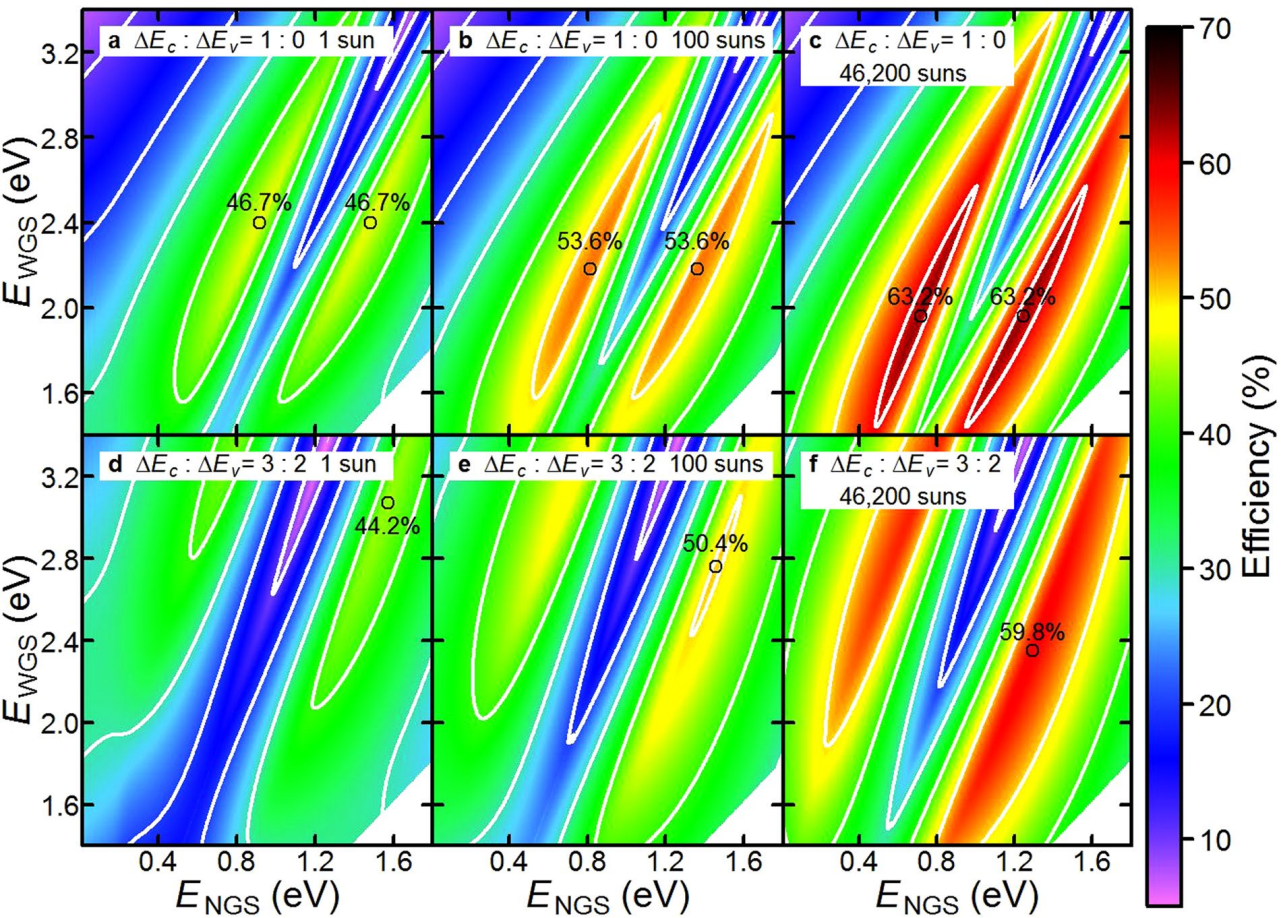
Two-dimensional counter maps of efficiency as functions of *E*_WGS_ and *E*_NGS_ under one-sun, 100 suns, and 46,200 suns. The black circles indicate the points at which the efficiencies are maximum.

**Figure 6:**
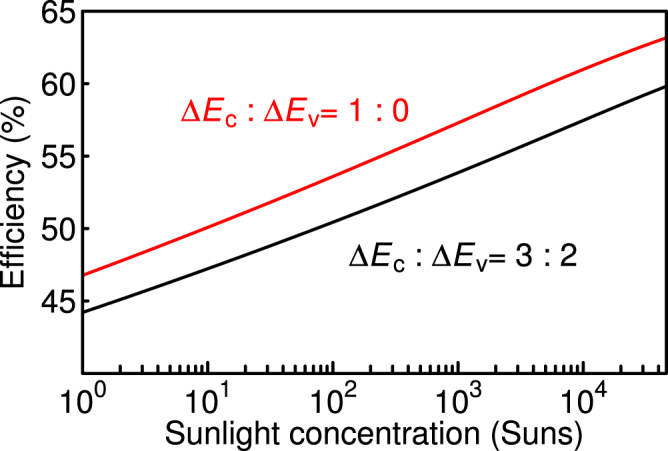
Maximum conversion efficiency as a function of sunlight concentration.

Figure 5a–f present the two-dimensional counter maps of the calculated efficiencies as functions of *E*_WGS_ and *E*_NGS_. Figure 5a–c present the results when Δ*E*_v_ is zero under one-sun, 100 suns, and maximum concentration, and Fig. 5d–f present the results at a Δ*E*_c_-to-Δ*E*_v_ ratio of 3:2. Under the maximum concentration (one-sun), the maximum conversion efficiencies for Δ*E*_c_-to-Δ*E*_v_ ratios of 1:0 and 3:2 are 63.2 (46.8) and 59.8 (44.2)%, respectively. The values of *E*_WGS_ and *E*_NGS_ which exhibit the maximum efficiency are 2.40 and 1.48 (or 0.92) eV (3.08 and 1.57 eV) for a Δ*E*_c_-to-Δ*E*_v_ ratio of 1:0 (3:2) under one-sun illumination, respectively. With the increase in voltage loss caused by the increase in Δ*E*_v_, the maximum efficiency tends to appear at a wider *E*_WGS_, attaining a higher voltage boost. According to the calculated results shown in Fig. 5, the optimum combination of the bandgaps can be covered by using, for example, InAlGaP or AlGaAs for the WGS and GaInNAs or GaBiNAs for the NGS. These materials can match with the lattice constant of GaAs. The bandgaps of these materials within the lattice match condition with GaAs are in the range of 1.4–2.2 eV for InAlGaP^25^, 1.4–2.1 eV for AlGaAs^26^, 0.8–1.4 eV for GaInNAs^27–29^, and 0.4–1.4 eV for GaBiNAs^30^. It is noted that the band discontinuity depends on the material system. Even if the combination of the bandgaps comprising the hetero-interface is suitable for the TPU-SC, the conversion efficiency is also influenced by the band discontinuity. Figure 6 shows the maximum conversion efficiency as a function of sunlight concentration. The solid and dashed lines indicate the efficiencies for the TPU-SCs with Δ*E*_c_-to-Δ*E*_v_ ratios of 1:0 and 3:2, respectively. According to these calculations, approximately 10 (80) equivalent suns are necessary to surpass an efficiency of 50% with the Δ*E*_c_-to-Δ*E*_v_ ratio of 1:0 (3:2).

## Discussion

The TPU-SC is one practical realisation of the idea of photon ratchet^21–23^. Therefore, conceptually, the TPU-SC and the ratchet-band IBSC are the same. However, in the band diagram, there exists a difference between the TPU-SC and the ratchet-band IBSC. For the ratchet-band IBSC, the output voltage corresponds to the quasi-Fermi splitting between electrons in the CB and holes in the VB of the host material. On the other hand, the output voltage of TPU-SC corresponds to the difference of quasi-Fermi levels between electrons in the CB of WGS and holes in the VB of NGS. This difference appears as a difference in the calculation framework. For the ratchet-band IBSC, the relationship among the bandgap of the host material, *E*_g_, the energy difference from the VB to IB, *E*_VI_, from the ratchet band to CB, *E*_RC_, and the IB to ratchet band, Δ*E*, is as follows:

12 $${E}_{{\rm{g}}}={E}_{{\rm{VI}}}+{E}_{{\rm{RC}}}+{\rm{\Delta }}E.$$

Equation (12) is corresponds to Eq. (5) for the TPU-SC, and *E*_g_, *E*_VI_, *E*_RC_, and Δ*E* correspond to *E*_WGS_, *E*_NGS_, Δ*E*_c_, and Δ*E*_v_, respectively. Here, Δ*E* in Eq. (12) must be negative because photo-generated electrons in the IB is necessary to relax towards the ratchet band. Conversely, for the TPU-SC, Δ*E*_v_ in Eq. (5) is better to be positive because photo-generated holes in the WGS is easy to drift towards the NGS.

The hetero-interface that boosts the voltage in a TPU-SC is separated from the portion generating additional current by absorbing below-gap photons in the WGS. This structure prevents energy relaxation from the CB of the WGS to that of the NGS, playing a similar role to that of the IB. Excited electrons and holes in the NGS are promptly separated in the internal electric field. Thereby, long-lived, high-density electrons accumulated in the depletion layer formed at the hetero-interface enables effective intraband excitation and accomplishes efficient TPU. The effect of the voltage boost decreases with Δ*E*_v_. Therefore, the conversion efficiency of the TPU-SC does not exceed the ideal value of the IBSC. However, the TPU-SC does not require any complicated quantum structures and is a SJSC containing a simple hetero-interface. Thus, we can expect bulk quality carrier transport in TPU-SCs. TPU-SCs have similar structure to dual-junction SCs. Dual-junction solar cells comprise a series of *p–n* junctions connected through a tunnelling junction, whereas a TPU-SC is a SJSC. Thus, TPU does not occur in a dual-junction SC, and its conversion efficiency is smaller than that of a TPU-SC, as expected in Fig. 6. Moreover, current matching between the WGS and NGS is not required in TPU-SCs. This advantage causes TPU-SCs to be robust against changes in the sunlight spectrum^31,32^. These results observed in the TPU-SC offer a route to high-conversion-efficiency solar cells exceeding 50%.

## Methods

We performed calculations using Visual Studio Community 2015 and GNU Scientific Library. The programming language used was Visual C++.

### Code availability

The computer code used in this study are available from the corresponding author upon request.

### Data Availability

The data that support the findings of this study are available from the corresponding author upon request.
